# *In Planta* Transcriptome and Proteome Profiles of *Spongospora subterranea* in Resistant and Susceptible Host Environments Illuminates Regulatory Principles Underlying Host–Pathogen Interaction

**DOI:** 10.3390/biology10090840

**Published:** 2021-08-28

**Authors:** Sadegh Balotf, Richard Wilson, Robert S. Tegg, David S. Nichols, Calum R. Wilson

**Affiliations:** 1 Tasmanian Institute of Agriculture, New Town Research Laboratories, University of Tasmania, New Town, TAS 7008, Australia; sadegh.balotf@utas.edu.au (S.B.); robert.tegg@utas.edu.au (R.S.T.); 2 Central Science Laboratory, University of Tasmania, Hobart, TAS 7001, Australia; richard.wilson@utas.edu.au (R.W.); d.nichols@utas.edu.au (D.S.N.)

**Keywords:** *Spongospora subterranea*, potato, *in planta* analysis, transcriptomics, proteomics

## Abstract

**Simple Summary:**

Infections of potato tubers and roots by *Spongospora subterranea* result in powdery scab and root diseases. Losses due to infections with *S. subterranea* are substantial in most potato-growing regions of the world with no fully effective treatments available. Understanding the gene regulation of pathogens in their host is dependent on multidimensional datasets. In this study, we profiled the transcriptome and proteome of *S. subterranea* within the susceptible and resistant host. Enzyme activity and nucleic acid metabolism appear to be important to the virulence of *S. subterranea*. Our results provide a good resource for future functional studies of powdery scab and might be useful in *S. subterranea* inoculum management.

**Abstract:**

*Spongospora subterranea* is an obligate biotrophic pathogen, causing substantial economic loss to potato industries globally. Currently, there are no fully effective management strategies for the control of potato diseases caused by *S. subterranea*. To further our understanding of *S. subterranea* biology during infection, we characterized the transcriptome and proteome of the pathogen during the invasion of roots of a susceptible and a resistant potato cultivar. A total of 7650 transcripts from *S. subterranea* were identified in the transcriptome analysis in which 1377 transcripts were differentially expressed between two cultivars. In proteome analysis, we identified 117 proteins with 42 proteins significantly changed in comparisons between resistant and susceptible cultivars. The functional annotation of transcriptome data indicated that the gene ontology terms related to the transportation and actin processes were induced in the resistant cultivar. The downregulation of enzyme activity and nucleic acid metabolism in the resistant cultivar suggests a probable influence of these processes in the virulence of *S. subterranea*. The protein analysis results indicated that the majority of differentially expressed proteins were related to the metabolic processes and transporter activity. The present study provides a comprehensive molecular insight into the multiple layers of gene regulation that contribute to *S. subterranea* infection and development *in planta* and illuminates the role of host immunity in affecting pathogen responses.

## 1. Introduction

The obligate biotrophic *Spongospora subterranea* is an important pathogen of potato, the world’s third most valuable food crop for human consumption. The pathogen induces root and tuber diseases in potato, the latter known as powdery scab, that impact both tuber yield and quality [1,2]. Currently, there are no effective strategies for the control of these root and tuber diseases. Thus, an understanding of the regulatory principles underlying *Spongospora*-potato interactions is important, particularly during colonization of potato roots [3]. *Spongospora subterranea* is a soil-dwelling obligate biotroph. Similar to other plasmodiophorids, *S. subterranea* has a complex lifestyle that allows the pathogen to persist in soil between host plants with the production of long-life resting spores [4]. In most genera of plasmodiophorids, resting spores are found as aggregates known as sporosori [5]. The resting spores germinate releasing short-lived zoospores in the presence of potato plants. Following attachment and encystment to a host root, the zoospore transfers its cellular contents to a host cell. The pathogen forms a plasmodium within infected cells which develops into a zoosporangium producing and releasing secondary zoospores that will induce further root and tuber infections. Zoospores are the main infective form of *S. subterranea* that can swim in the soil and search for the potato roots to infect with a specialized extrusome and develop a zoosporangium [6]. This zoospore-mediated root infection can form root galls approximately 1–3 months following the infection, with galls producing sporosori that can be released into the soil as roots degrade following senescence [7].

RNA-sequencing has been widely used to investigate the molecular aspects of plant–pathogen interaction with more focus on the host responses during infection with pathogens [8,9,10]. A deep sequencing approach allows the simultaneous capture of both plant and pathogen transcripts during infection [11]. The transcriptome analysis of the necrotrophic fungus *Bipolaris sorghicola* inside the sorghum leaves revealed the key genes in the plant–pathogen interaction that included transcriptional factors and plant cell-wall degrading enzymes [12]. Teixeira et al. [13] profiled the transcriptome of *Moniliophthora perniciosa* in the infected shoots of the *Theobroma cacao* and identified putative virulence effectors by characterizing the fungal infection strategies. The *in planta* transcriptome analysis of the bacterial pathogen *Pseudomonas syringae* in Arabidopsis revealed specific “immune-responsive” bacterial genes and processes [14]. A time-course analysis of the rusty root rot pathogen *Ilyonectria robusta* transcriptome *in planta* revealed pathogenicity-related genes, in particular candidate effector genes [15]. Although transcriptome analysis is a useful approach for the study of plant–pathogen interaction, it has been well established that transcript levels do not always reflect protein levels and protein activities. Mass spectrometry (MS) based proteomics has previously been applied to pathogens in vitro and *in planta*. Previous *in planta* proteomics analyses of the biotrophic barley fungal pathogen *Blumeria graminis* identified several proteins related to pathogenicity inside the infected tissues [16]. The proteome analyses of proteases in the biotroph fungal pathogen *Cladosporium fulvum* confirmed the presence of six proteases at proteome level during host infection [17]. However, the capacity of proteomics to describe pathogens *in planta* remains limited as the pathogen proteins are diluted by the plant proteome. Therefore, the integration of transcriptomics and proteomics is essential to acquire a precise picture of pathogens during the colonization of their host.

To understand the biology and virulence of obligate biotrophic plant pathogens, a comprehensive analysis of the transcriptome and proteome of these pathogens is a priority. We have researched the transcriptome [18] and proteome [19] dynamics of *S. subterranea* during the germination of resting spores using an in vitro model. However, to better understand the complex life cycle of the obligate biotrophic pathogens inside their host, a comparable effort to analyse the *in planta* transcriptome and proteome of *S. subterranea* during root infection is required. To unravel key components of the infection and interaction with the host during the biotrophic interaction, we simultaneously profiled the transcriptome and proteome of *S. subterranea* inside the potato roots of a susceptible and a resistant potato cultivar. We also compared the *in planta* data to our previous in vitro analysis of *S. subterranea* transcriptome and proteome during the germination of spores.

## 2. Materials and Methods

### 2.1. Pathogen Source and Potato Cultivars Used in This Study

Two potato cultivars that differ in resistance to *S. subterranea* were used in this study. Cultivar ‘Gladiator’ has a strong resistance to both tuber and root disease, while cultivar ‘Iwa’ is known as being highly susceptible to root and tuber infection by *S. subterranea* [6]. The *S. subterranea* inoculum was obtained by dry scraping lesions from powdery scab-infected tubers and purified using Ludox^®^ (HS-40 colloidal silica, Sigma, NSW, Australia) as described before [20].

### 2.2. Plant Growth and Pathogen Infection

Potato plants obtained from single-node cuttings were grown in sterile tissue culture in standard Murashige and Skoog (MS) medium with 30 g/L, 500 mg/L of casein hydrolysate and 40 mg/L of ascorbic acid [21]. During the initial growth stage, plants were maintained under a 16 h light/8 h dark photoperiod at 22 °C. After three weeks of culture, seedlings were carefully uprooted from their medium and inoculated by dipping their roots in an inoculum suspension (two milligrams of dried *S. subterranea* resting spore in 2 mL of Hoagland solution, incubated at 25 °C for 3 days) for one hour. The seedlings of similar size were then transplanted into plastic pots filled with wetted sterilized potting mix and transferred to a greenhouse with a relative humidity of 80 ± 5% and a temperature of 25 ± 3 °C. To ensure strong disease pressure, an additional 20 mL of the inoculum suspension (one gram of dried *S. subterranea* resting spore in 1 L of water) was added directly to the potting mix fourteen days after planting. After 6 weeks in the greenhouse, when *Spongospora* root galls were visible on the roots, potato roots were collected for RNA-seq (*n* = 3) and proteome (*n* = 4) analysis. The collected roots were washed thoroughly under running water, frozen in liquid nitrogen and stored at −80 °C until use.

### 2.3. RNA Extraction and Sequencing

Total RNA was extracted from 50 mg of each sample using the RNeasy plus mini kit (Qiagen, Hilden, Germany) including the gDNA eliminator spin columns to remove genomic DNA. RNA concentration was checked by a Qubit fluorometer using the Qubit RNA Broad-Range kit (Invitrogen, Waltham, MA, USA). The integrity of the RNA samples was analyzed using an Agilent 2100 Bioanalyzer system (Agilent, Palo Alto, Santa Clara, CA, USA). A total of 4 μg of RNA per sample was used as the input material for further analysis. The Illumina TruSeq Stranded Total RNA Library Prep Kit with RiboZero (Illumina, San Diego, CA, USA) was used to produce the sequencing libraries that were then sequenced on the NovaSeq6000 (Illumina, San Diego, CA, USA) platform (50 M, 2 × 100 bp) at the Australian Genome Research Facility (AGRF, Melbourne, Victoria, Australia).

### 2.4. Transcriptome Assembly and Differential Expression Analysis

The transcriptome analysis was performed using RNA-seq analysis tools available on the Galaxy Australia platform (https://usegalaxy.org.au/ (accessed on 5 February 2021)). Paired-end reads were initially quality checked using FastQC v.0.11.9 tools. Reads of low quality (<20 nucleotides) and of adapter contaminations were trimmed by Trimmomatic v.0.36.6. The quality of trimmed reads was assessed again using FastQC. The processed reads were mapped to the host first (the potato reference genome, downloaded from http://solanaceae.plantbiology.msu.edu/pgsc_download.shtml (accessed on 5 February 2021)) with the remaining reads mapped to the *S. subterranea* draft genome [22] using the HISAT2 v.2.1.0 tool. Stringtie 2.1.1 was used for *de novo* transcriptome reconstruction of the aligned reads. The Stringtie-Merge tool was then used to combine the Stringtie outputs and create a transcriptome database. To determine the uniquely mapped reads, FeatureCounts v.2.0.1 was used to generate a count matrix by calculating the transcript’s abundance using the fragments per kilobase of per million mapped reads (FPKM). The mapped reads were then served as input to DESeq2 v.2.11.40.6 tool for the quantification of differential gene expression. The list of differentially expressed genes (DEGs) was filtered with a cut-off for a false discovery rate (FDR) of 0.05. The RNA-seq data were further validated using qRT-PCR as described before [18]. The primer sequences were listed in Supplementary Table S1.

### 2.5. Protein Extraction and Digestion 

Proteins were extracted from 50 mg of frozen roots per sample by adding 150 µL of extraction buffer including 7 M urea and 2 M thiourea, 1% dithiothreitol (DTT), 100 mM NaCl, 40 mM Tris, pH 8.0 and protease inhibitor cocktail (cOmplete Mini EDTA-free; Roche Diagnostics, NSW, Australia). The mixture was homogenized two times for 60 s each using a Fast Prep-24 bead beater (Mp Biomedicals, Seven Hills, NSW, Australia) at 5500× *g* in room temperature. Samples were then centrifuged at 16,000× *g* for 10 min at 4 °C and the supernatant was transferred to a new tube. Six volumes of cold acetone were added to the resulting clarified extracts and tubes incubated at −20 °C overnight. The tubes were centrifuged at 10,000× *g* for 8 min, the protein pellets were left to air dry for 5 min at room temperature and resuspended in denaturing buffer (7 M urea and 2 M thiourea, 40 mM Tris, pH 8.0 and protease inhibitor). Protein concentration was estimated by a Qubit fluorometer using the Qubit protein assay kit (Thermo Fisher Scientific, Waltham, MA, USA). Subsequently, DTT was added to a final concentration of 10 mM and proteins were reduced overnight at 4 °C. Samples were then alkylated in dark using 50 mM iodoacetamide for 2 h at room temperature and then digested with trypsin/LysC (Promega, Madison, WI, USA) according to the SP3 method [23]. Peptides were desalted using ZipTips (Merck, Darmstadt, Germany) and dried down in a SpeedVac concentrator.

### 2.6. Data-Independent Acquisition Mass Spectrometry (DIA-MS)

Peptide samples were dissolved in 12 µL loading buffer (0.05% (*w/w*) trifluoroacetic acid in water/acetonitrile (98:2)). Approximately 1 μg of each sample was analyzed using an Ultimate 3000 nano RSLC system (Thermo Fisher Scientific, Waltham, MA, USA) coupled to a Q-Exactive HF mass spectrometer fitted with nanospray Flex ion source (Thermo Fisher Scientific, Waltham, MA, USA) and controlled using Xcalibur version 4.3. Peptides were preconcentrated onto a 2 cm PepMap 100 C18 trap column at a flow rate of 5 µL/min and then separated at 300 nL/min on a 25 cm PepMap 100 C18 analytical column held at 45 °C using a 120-min segmented gradient. Mobile phase A comprised 0.1% (*w/w*) formic acid in water and mobile phase B comprised 0.08% (*w/w*) formic acid in acetonitrile/water (80:20). Data acquisition parameters were: 2.0 kV spray voltage, S-lens RF level of 60 and heated capillary set to 250 °C. MS1 spectra (390–1240 *m*/*z*) were acquired at a scan resolution of 120,000 in profile mode with an AGC target of 3e6 and followed by sequential MS2 scans across 26 DIA × 25 amu windows over the range of 397.5–1027.5 *m*/*z*, with 1 amu overlap between sequential windows. MS2 spectra were acquired in centroid mode at a resolution of 30,000 using an AGC target of 1 × 10^6^, maximum IT of 55 ms and normalized collision energy of 27.

### 2.7. Raw Data Processing and Protein Label-Free Quantitation (LFQ)

DIA-MS raw files were processed using Spectronaut software (ver 14.8) (Biognosys AB, Wagistrasse, Switzerland). A project-specific library was generated using the Pulsar search engine to search the DIA MS2 spectra against the *Solanum tuberosum* UniProt reference proteome (UP000011115) comprising 53,106 entries concatenated with the UniProtKB protein sequences for *S. subterranea* (11,129 entries). For library generation, N-terminal acetylation and methionine oxidation were included as variable modifications, and cysteine carbamidomethylation was specified as a fixed modification and up to two missed cleavages were allowed. Peptide, protein, and PSM thresholds were set to 0.01. Mass tolerances were based on first pass calibration and extensive calibration for the calibration and main searches, respectively, with correction factors set to 1 at the MS1 and MS2 levels. Targeted searching of the library based on XIC extraction deployed dynamic retention time alignment with a correction factor of 1. Protein identification deployed 1% q-value thresholds at the precursor and protein levels, and automatic generation of mutated peptide decoys based on 10% of the library and dynamic decoy limitation for protein identification. MS2-level data were used for relative peptide quantitation between experimental samples, using the intensity values for the Top3 peptides (stripped sequences) and cross-run normalization based on median peptide intensity. Proteins identified on the basis of a single peptide (single hit proteins) were excluded while only proteins identified by matching to entries in the *S. subterranea* database were included in further analysis.

### 2.8. Gene Ontology Enrichment Analysis

The identified transcripts were subject to BLAST analysis against the UniProt (www.uniprot.org (accessed on 25 April 2021)) and NCBI (non-redundant protein and non-redundant transcript) databases with an e-value cut-off of 10^−5^. For the differentially expressed transcripts and proteins, gene ontology enrichment and pathway analysis were derived from UniProt, DAVID and the Kyoto Encyclopedia of Genes and Genomes (KEGG) databases (www.genome.jp/kegg (accessed on 10 May 2021)). The PCA plot and heatmap were drawn using Perseus software (v. 1.6.14.0). The volcano plot was obtained from the VolcaNoseR app [24].

## 3. Results and Discussion

### 3.1. In Planta Transcriptome and Proteome Profiling of S. subterranea

**Figure 1:**
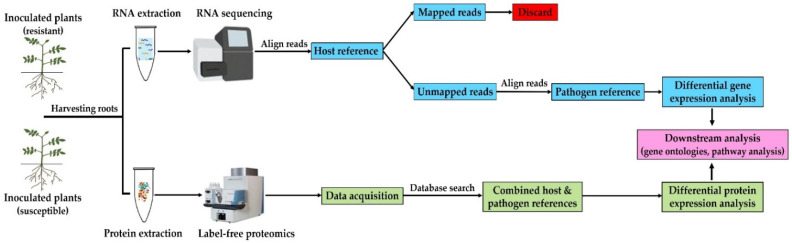
Workflow of analysis. Transcriptome and proteome of *S. subterranea* were analyzed simultaneously by using different approaches. The mixed transcriptome obtained from *S. subterranea*-infected potato roots was sequenced by using Illumina mRNA-Seq technology. The reads were aligned to the potato reference genome and unaligned reads were used to analyse the transcriptome of *S. subterranea*. For the proteome analysis, proteins were extracted from *S. subterranea*-infected potato roots, analyzed using a label-free approach and searched in a combined host and pathogen database. Further analysis using multiple bioinformatics approaches identified the functional annotation of the differentially expressed genes and proteins.

In this study, we have employed RNA-seq and label-free proteomics approaches to profile the transcriptome and proteome of *S. subterranea* inside potato roots of disease resistant (cv. Gladiator) and susceptible (cv. Iwa) cultivars. The experimental workflow for the RNA and protein preparation and omics data analysis is summarized in Figure 1.

Applying these approaches for the *in planta* transcriptome and proteome analysis of *S. subterranea* enabled the identification of 7650 transcripts and 117 proteins, respectively (Table 1). The lower number of identified proteins can be explained by the fact that the *in planta* protein analysis of pathogens is technically challenging as the pathogen proteins are embedded in and diluted by the plant proteome. There are published reports that analyzed the *in planta* proteome of pathogens, but only a very limited number of pathogen proteins have been identified [16,25].

### 3.2. Transcriptome Profile of S. subterranea inside the Resistant and Susceptible Hosts

**Figure 2:**
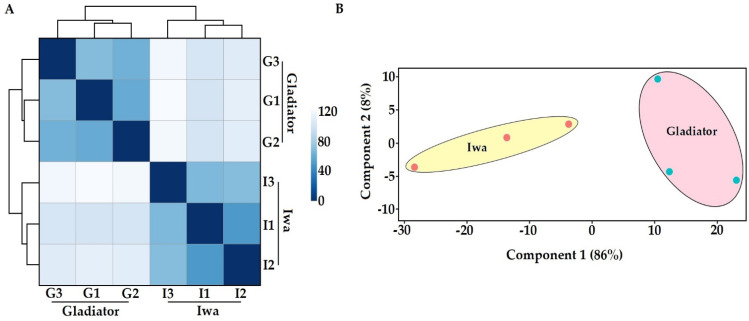
Hierarchical clustering of the six samples used in RNA-seq study showing two distinct clades (**A**) and the principal component analysis of RNA-seq data (**B**). G; Gladiator, I; Iwa.

To analyse the transcriptome of the obligate biotrophic pathogen *S. subterranea* in potato roots, a deep sequencing approach with an rRNA removal (to enrich mRNA) strategy was employed to overcome the RNA quantity difference between the host plant and pathogen. This method successfully enriched pathogen sequences allowing us to profile high-quality *S. subterranea* transcriptomes *in planta* with more than 4 million mean reads using the Illumina HiSeq platform (Supplementary Table S2). The transcriptome results were validated by qPCR measurements of the selected DEGs with a correlation coefficient above 88% (Supplementary Figure S1), indicating the accuracy of our RNA-seq data. Of 7650 identified unigenes, 6458 unigenes (~85%) matched the non-redundant (NR) protein and UniProt databases, suggesting that the de novo assembly approach was reliable. The complete list of identified and annotated transcripts is provided as Supplementary Table S3. The number of identified genes from *S. subterranea* in this study exceeded what has been reported in other *in planta* transcriptome analysis of plant pathogens [12,26,27]. According to the hierarchical clustering (sample-to-sample distances) of all six samples (Figure 2A) and principal component analysis (PCA) (Figure 2B), our RNA-seq approach was sensitive enough to capture *S. subterranea* transcriptome differences between the biological replicates.

### 3.3. Functional Annotation of Differently Expressed Genes 

**Figure 3:**
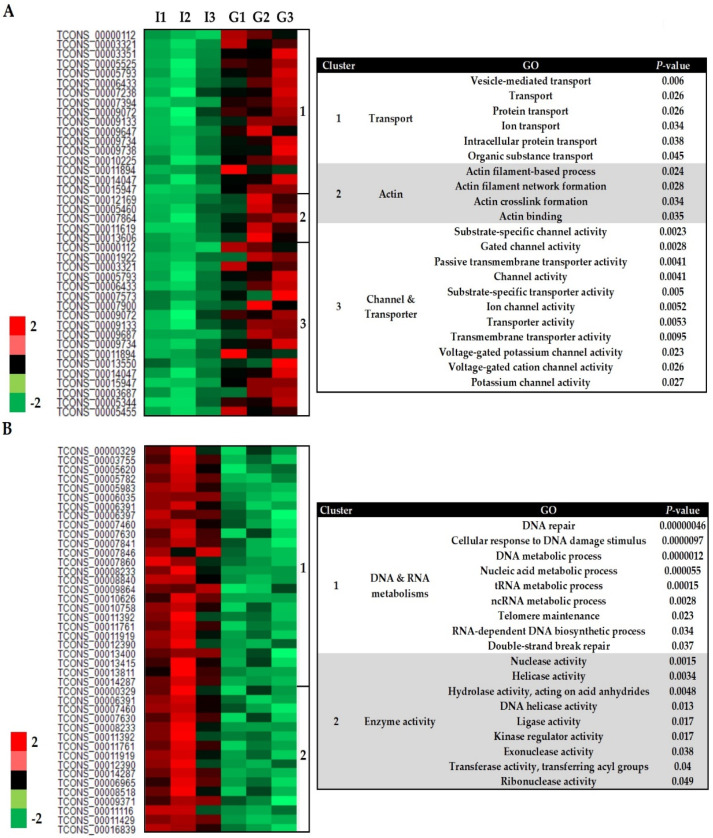
Heatmap (based on Z-scores of the normalized expression of transcripts) of differently expressed genes from *S. subterranea* (Gladiator vs. Iwa). The upregulated (**A**) and downregulated (**B**) genes and their related GO terms. G; Gladiator, I; Iwa.

A total of 1151 genes changed significantly in abundance (FDR < 0.05) in the transcriptome of *S. subterranea* (Gladiator vs. Iwa), including 837 upregulated and 314 downregulated genes (Supplementary Figure S2). Our gene ontology (GO) analysis revealed five major sub-clusters (three up and two downregulated classes) among the DEGs. The Z-scored expression levels of these DEGs were represented in the heatmap (Figure 3). The upregulated DEGs fell into three main clusters including transport (17 genes, 6 GO terms), actin (5 genes, 4 GO terms) and channel/transporter (18 genes, 11 GO terms) (Figure 3A). Upregulation of processes related to transportation and actin emphasized the role of ion exchange and structural rearrangement of the actin cytoskeleton in the pathogen during communication with hosts [28,29]. A transporter is a type of transmembrane protein that absorbs essential nutrients using the energy from ATP hydrolysis and can be divided into importers and exporters [30]. Transporter activity has been associated with spore germination and pathogenicity of *Schizosaccharomyces pombe* [31], *Botrytis cinerea* [32], *Colletotrichum gloeosporioides* [33], and *Fusarium graminearum* [34]. However, our RNA-seq data provide the first evidence that transporter and channel activities are also involved in *Spongospora*–potato interaction and potentially during the host infection.

The downregulated DEGs formed two main clusters: DNA/RNA metabolism (26 genes, 9 GO terms) and enzyme activity (16 genes, 9 GO terms) (Figure 3B). One of the major significantly over-represented groups in these DEGs encoded for proteins involved in DNA and RNA metabolism (Figure 3B). The successful germination of dormant spores needs the progressive re-starting of transcription and translation [35]. To accelerate the transcription processes during the early stages of germination, DNA relaxes rapidly from the supercoiled state. This requires the activity of helicase enzymes and DNA repair genes [36,37]. The spores deficient in DNA repair in *Bacillus subtilis* were delayed in the first division of the spore′s nucleoid and severely affected by a ROS-inducer during the germination [38]. Thus, the resistant cultivar, Gladiator, might suppress the germination of *S. subterranea* resting spores by downregulation of the biological process related to nucleic acid metabolism such as transcription and DNA repair. The transfer RNA (tRNA) metabolic process is another GO term associated with the downregulated DEGs (Figure 3B). The tRNA pathway is an alternative route for cytokinin biosynthesis in many organisms [39]. Plant pathogens have developed several strategies to prevail over their hosts including the production of phytohormones such as cytokinin which interfere with host immune responses [40]. The tRNA-mediated cytokinin production by the rice blast fungus *Magnaporthe oryzae* reduces the defence responses from the host and alters the amino acid and sugar distribution to the favour of a pathogen [41]. The hemibiotroph *Colletotrichum graminicola* altered cytokinin responses in maize leaves, either through fungal production of cytokinin or using an indirect route [42]. Cytokinin production contributes to pathogen virulence of the plant pathogen *Claviceps purpurea* by the induction of susceptibility [43]. Therefore, the resistant cultivar can limit the access of *S. subterranea* to the plant amino acid and sugar sources by downregulation of the tRNA metabolic process. Hence, our results could lay a foundation for further investigations into the influences of nucleic acid metabolism in potato susceptibility to powdery scab.

Several enzymes including members of the hydrolase, kinase, transferase, ligase, and helicase families were downregulated in the *S. subterranea* transcriptome in the resistant cultivar (Figure 3B). Hydrolase (EC3) is a class of enzymes that catalyse the cleavage of a covalent bond using water and are known to participate in the germination of spores in fungal and bacterial pathogens by facilitating the reconstruction of the cell wall [44,45]. These enzymes were shown to be involved in the pathogen invasion and pathogenicity of *Colletotrichum lagenarium* [46] and *Cladosporium fulvum* [47]. Thus, the downregulation of hydrolase enzymes in the transcriptome of *S. subterranea* inside the resistant cultivar might be related to their role in the germination of resting spores as well as *S. subterranea* pathogenicity [48]. The protein kinases (EC2) that catalyse the modification of proteins by phosphorylation were another downregulated enzyme group (Figure 3B). The role of protein kinases in the resting spore germination have been confirmed in *Verticillium dahlia* [49], *Bacillus subtilis* [50], *Schizosaccharomyces pombe* [51], and many other pathogens [52]. Consistent with the above results, our RNA-seq analysis confirmed the downregulation of several enzyme families in the transcriptome of *S. subterranea* inside the resistant cultivar compared to the susceptible one. Although not enough is known about the *Spongospora*-potato interaction during the root infection, our RNA-seq data suggest that suppression of enzyme activity, as well as nucleic acid metabolism, can contribute to resistance against *S. subterranea*.

### 3.4. In Planta Protein Analysis of S. subterranea

**Figure 4:**
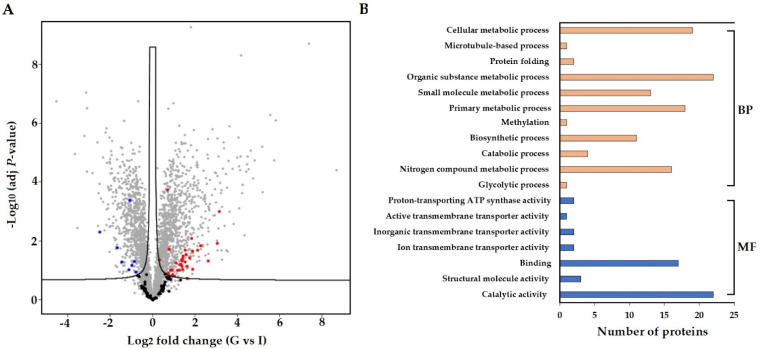
Comparison of differently abundant proteins (FDR < 0.05) in resistant and susceptible potato cultivars. Proteins from *S. subterranea* are bolded (black; no significant, red; upregulated and blue downregulated) (**A**). The GO term for the *S. subterranea* DEPs (**B**). BP; biological process, MF; molecular function, G; Gladiator, I; Iwa.

A label-free proteomics approach was used to profile the proteome of *S. subterranea* in the infected roots of potato cultivars. A total of 117 proteins from *S. subterranea* were identified in which 42 proteins were differentially expressed between Gladiator and Iwa (Figure 4A). A complete list of all identified and DEPs (Gladiator vs. Iwa) is provided as Supplementary Table S4. The GO analysis (biological process and molecular function) of the total identified and DEPs is shown in Figure 4B. This analysis revealed that DEPs were mainly concentrated on the catabolic and metabolic processes, binding, nitrogen compound metabolic process, and biosynthetic process. The proteins related to the metabolic and catabolic processes are very abundant soluble proteins and are generally well represented in several pathogens such as *Phytophthora capsici* [53], *Colletotrichum acutatum* [54], and *Botrytis cinerea* [55]. Similar to the RNA-seq results, the existence of few molecular functions related to “transporter activity” such as ion transmembrane transporter activity, inorganic transmembrane transporter activity, active transmembrane transporter activity and proton-transporting ATP synthase activity was confirmed in the proteomics study (Figure 4B). Plants have developed a high capacity to recognize pathogens through various strategies and pathogens manipulate the defence response through secretion of virulence effectors [56]. Successful pathogens can suppress the plant responses and thereby cause disease. This suppression is achieved through the deployment of the effector proteins. However, plants are able to recognize the pathogen effectors proteins through an accessory protein or by direct physical association [57]. We identified 34 upregulated and eight downregulated proteins in the resistant cultivar compared to the susceptible cultivar (Figure 4A). The activation of several metabolic and catabolic processes as well as transporter activity was also confirmed among the DEPs (Figure 4B). Thus, we concluded that *S. subterranea* requires an intensive effort for the suppression of the host immune system in Gladiator which results in a more active plant–pathogen interaction. Unfortunately, 20 proteins out of 42 DEPs were not annotated in databases and presented as “uncharacterized protein” (Supplementary Table S4). The genome annotation is not complete in plasmodiophorids and many of the proteins associated with spore germination and disease development have yet to be characterized in *S. subterranea*. We believe that the full implication of our present protein analysis will be realised when functional annotation of these DEPs is available.

**Figure 5:**
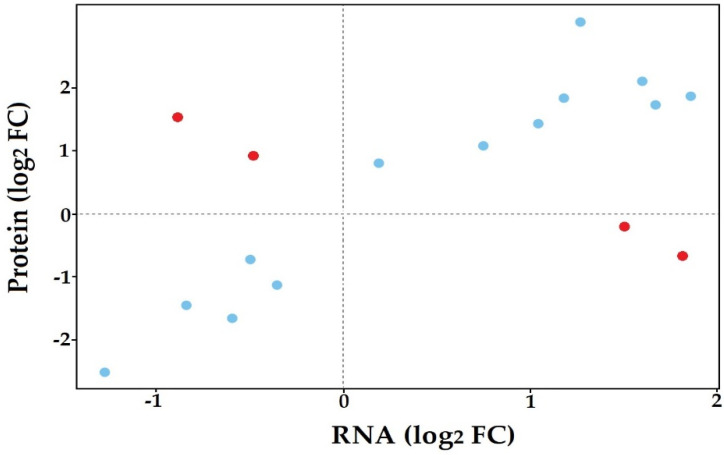
Distribution of proteins common between transcriptomics and proteomics experiments.

Comparison of the *in planta* transcriptome and proteome of *S. subterranea* identified 17 proteins in common between both datasets (Supplementary Table S5). We then plotted the log-transformed fold changes of proteins that were presented in RNA-seq and proteomics data (Figure 5). A high degree of concordance in changes in protein abundance between the two datasets was observed, with only four proteins having opposing changes in abundance between the RNA-seq and proteomics data (red dots in Figure 5). The MS/MS raw data were also searched against the proteome dataset generated from our RNA-seq data based on six-frame translation. A total of 44 proteins were found in this search (Supplementary Table S6) which were categorized into three groups according to functional annotation and pathway analysis (Table 2). The presence of GO terms related to peptide metabolism and translation showed that these processes are vital for the successful germination *in planta* as well as disease invasion of *S. subterranea* inside the host. The dependence of spore germination on transcription and translation has been demonstrated in many pathogens [58,59,60]. This finding aligned with the observed upregulation of transcription and protein synthesis in our previous in vitro analysis of transcriptome and proteome of *S. subterranea* during the germination of resting spores [18,19]. Therefore, transcription and translation may be induced upon the activation of resting spores both *in planta* and in vitro.

### 3.5. Co-Expression of In Planta and In Vitro Analysis of S. subterranea Transcriptome and Proteome

**Figure 6:**
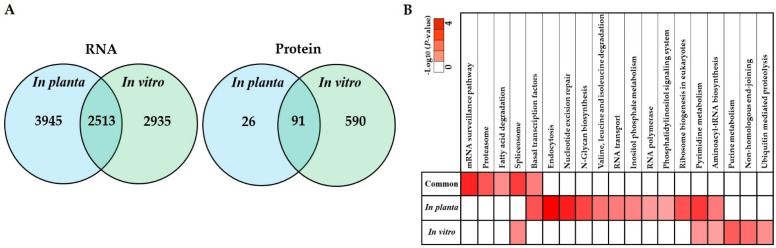
Venn diagrams depicting the overlap of identified proteins common between or independent in the in vitro and *in planta* analysis of *S. subterranea* transcriptome and proteome (**A**). Pathway analysis of identified proteins common between or independent in the in vitro and *in planta* analysis of *S. subterranea* transcriptome (**B**).

The *in planta* transcriptomes and proteomes of *S. subterranea* obtained in this study were compared to our previous in vitro transcriptome [18] and proteomic [19] studies of *S. subterranea*. The number of identified genes was significantly higher in the *in planta* analysis of *S. subterranea* transcriptome (Figure 6A, left). In contrast, the number of identified proteins in the in vitro analysis of *S. subterranea* proteome was higher compared to *in planta* (Figure 6A, right). The pathway analysis of identified proteins common between or independent in the in vitro and *in planta* analysis of *S. subterranea* transcriptome is presented in Figure 6B. The main pathways identified during this research include nucleotide excision repair, pyrimidine metabolism, transcription, translation, transport, protein degradation, pyrimidine biosynthesis and purine metabolism. The mRNA surveillance pathway, proteasome, fatty acid degradation, spliceosome and basal transcription factors were the prominent pathways in the common proteins between the in vitro and *in planta* transcriptome of *S. subterranea*. These pathways are related to transcription, translation and amino acid degradation processes [58,59]. The purine metabolism and ubiquitin-mediated proteolysis were produced in vitro only, and these pathways were not recovered *in planta* (Figure 6B). On the other hand, some pathways such as RNA transport, inositol phosphate metabolism and phosphatidylinositol signaling system were only recovered *in planta* [61]. In total, the co-expression of *in planta* and in vitro transcriptome analysis of *S. subterranea* revealed that the spore activation in both *in planta* and in vitro needed the activity of the mRNA synthetase, DNA repair, translation and carbohydrate metabolism [62,63,64].

## 4. Conclusions

In conclusion, our detailed *in planta* analysis of the *S. subterranea* transcriptome and proteome revealed broad-scale differences between susceptible and resistant potato cultivars. The upregulation of transporters/channels and actin metabolism in the resistant cultivar was discovered in this study. Downregulation of the enzyme activity and nucleic acid metabolism in *S. subterranea* transcriptome in the resistant cultivar is one of the most important findings of the present study. The suppression of these processes in *S. subterranea* within roots of the resistant cultivar provides new insight into *Spongospora*–potato interaction and may lead to the development of novel approaches for the resistance against powdery scab disease in potato breeding programs. The proteome data reported in this study provides the first *in planta* analysis of *S. subterranea* inside the potato roots and validates our transcriptome results. However, many of the identified proteins in the proteome analysis were not annotated according to publicly available databases. The value of this protein analysis will be understood when the genome annotation is completed in plasmodiophorids. In total, our study contributes to the significant progress in our understanding of the interaction between the obligate biotrophic pathogens and their host plants and increased the availability of “omic” data in such a complex interaction.

## Figures and Tables

**Table 1:** The number of identified and significantly changing (FDR < 0.05) transcripts and proteins from *S. subterranea* inside the resistant and susceptible hosts.

	Identified	Differentially Expressed (Gladiator vs. Iwa)
RNA	7650	1377
Protein	117	42

**Table 2:** GO term for the proteins from *S. subterranea* identified in the database created from translated transcripts from RNA-seq analysis.

Categories	GO Terms	adj *p*-Value
Biological process	Peptide metabolic process	0.0032
	Organonitrogen compound metabolic process	0.0032
	Cellular amide metabolic process	0.0032
	Translation	0.014
	Peptide biosynthetic process	0.014
	Amide biosynthetic process	0.014
Molecular function	Structural constituent of ribosome	0.00004
	Structural molecule activity	0.00009
Pathway	Ribosome	0.00048

## Data Availability

The original contributions presented in the study can be found here: the transcriptome data is deposited in the NCBI BioSample Submissions (SRA) repository, accession number PRJNA747755. The proteomics data have been deposited to the ProteomeXchange Consortium via the PRIDE [65] partner repository with the dataset identifier PXD027266.

## Supplementary Materials

The following are available online at https://www.mdpi.com/article/10.3390/biology10090840/s1, Figure S1: Correlation between RNA-seq and qPCR, Figure S2: Comparison of differently abundant transcripts from *S. subterranea* in resistant and susceptible potato cultivars, Table S1: List of primers used in this study, Table S2: RNA-seq information, Table S3: List of identified, annotated and differentially expressed transcripts, Table S4: List of identified and differentially expressed proteins, Table S5: List of common proteins in both RNA-seq and proteomics, Table S6: List of identified proteins in our transcriptome database.
