# Supplementary material for: In Planta Transcriptome and Proteome Profiles of Spongospora subterranea in Resistant and Susceptible Host Environments Illuminates Regulatory Principles Underlying Host–Pathogen Interaction
Source: Biology (Basel). 2021 Aug 28;10(9):840. doi: 10.3390/biology10090840 (PMC8471823; doi:10.3390/biology10090840)

Figure S1- Correlation between RNA-seq and qPCR. Ten randomly selected transcript were subjected to the qPCR analysis. The log-transferred of RNA-seq results and qPCR plotted in Figure S1.

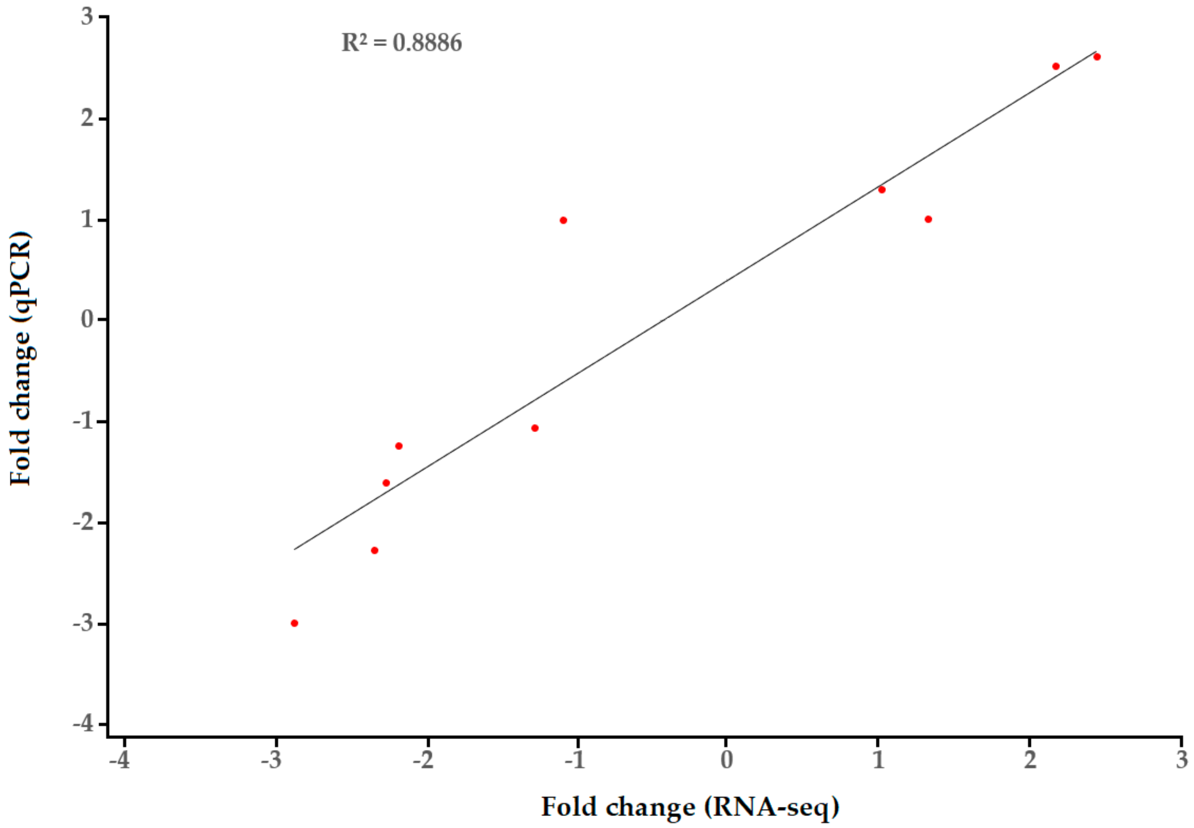

Supplement: Supplementary file 1 [file biology-10-00840-s001.zip › Figure S1- Correlation between RNA-seq and qPCR.pdf]
