# Supplementary material for: In Planta Transcriptome and Proteome Profiles of Spongospora subterranea in Resistant and Susceptible Host Environments Illuminates Regulatory Principles Underlying Host–Pathogen Interaction
Source: Biology (Basel). 2021 Aug 28;10(9):840. doi: 10.3390/biology10090840 (PMC8471823; doi:10.3390/biology10090840)

Figure S2- Comparison of differently abundant transcripts from *S. subterranea* in resistant and susceptible potato cultivars. G; Gladiator, I; Iwa

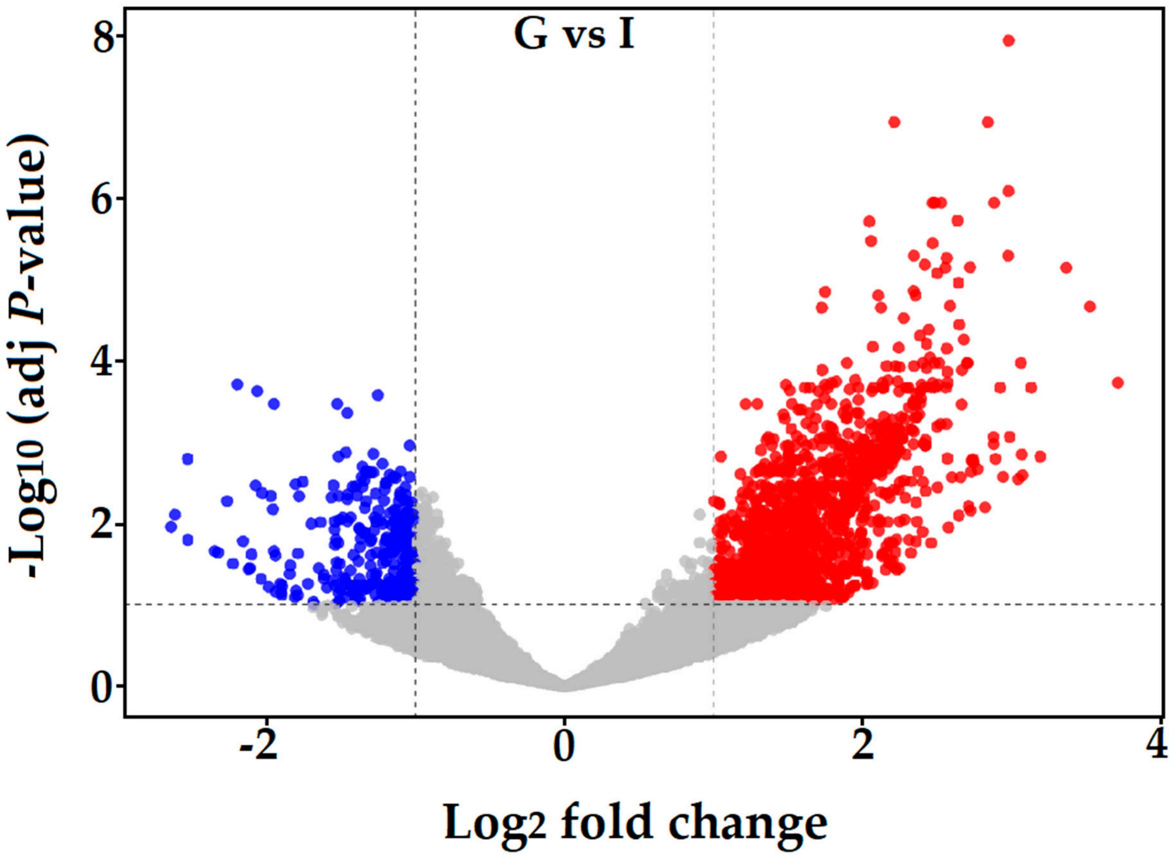

Supplement: Supplementary file 1 [file biology-10-00840-s001.zip › Figure S2- Comparison of differently abundant transcripts from S. subterranea in resistant and susceptible potato cultivars.pdf]
