# Supplementary material for: In Planta Transcriptome and Proteome Profiles of Spongospora subterranea in Resistant and Susceptible Host Environments Illuminates Regulatory Principles Underlying Host–Pathogen Interaction
Source: Biology (Basel). 2021 Aug 28;10(9):840. doi: 10.3390/biology10090840 (PMC8471823; doi:10.3390/biology10090840)
Supplement: Supplementary file 1 [file biology-10-00840-s001.zip › Table S2- RNA-seq information.pdf]

Table S2. RNA-seq raw reads and alignment statistics		
	Total quality reads	Mapped reads to Spongospora
G1	74,130,035	4,299,542
G2	59,956,347	2,326,306
G3	71,378,260	6,509,697
**Average Gladiator**	**68,488,214**	**4,378,515**
I1	60,499,120	3,623,897
I2	62,383,322	4,123,537
I3	71,612,245	4,253,767
**Average Iwa**	**64,831,562**	**4,000,400**
