# Supplementary material for: In Planta Transcriptome and Proteome Profiles of Spongospora subterranea in Resistant and Susceptible Host Environments Illuminates Regulatory Principles Underlying Host–Pathogen Interaction
Source: Biology (Basel). 2021 Aug 28;10(9):840. doi: 10.3390/biology10090840 (PMC8471823; doi:10.3390/biology10090840)
Supplement: Supplementary file 1 [file biology-10-00840-s001.zip › Table S5- List of common proteins in both RNA-seq and proteomics.pdf]

Table S5- List of proteins identified in both transcriptome and proteome analysis

No.	Accession Number	Annotation
1	A0A0H5R9V4	Uncharacterized protein
2	A0A0H5QQ41	Uncharacterized protein
3	A0A0H5R774	Uncharacterized protein
4	A0A0H5R6P1	Phosphoenolpyruvate carboxykinase (GTP) (EC 4.1.1.32)
5	A0A0H5RUC1	Uncharacterized protein
6	A0A0H5R7A6	Uncharacterized protein
7	A0A0H5RCH5	NodB homology domain-containing protein
8	A0A0H5R6J1	Uncharacterized protein
9	A0A0H5RJ90	Uncharacterized protein
10	A0A0H5QJF8	Uncharacterized protein
11	A0A0H5QLH5	Uncharacterized protein
12	A0A0H5QYG3	Uncharacterized protein
13	A0A0H5QSM4	GH16 domain-containing protein
14	A0A0H5R4K2	Uncharacterized protein
15	A0A0H5R4U4	Uncharacterized protein
16	A0A0H5R3K8	Uncharacterized protein
17	A0A0H5R3W2	Uncharacterized protein
